# Exposed Pulps—Treatment and Filling

**Published:** 1882-07

**Authors:** J. P. Wilson

**Affiliations:** Burlington


### EXPOSED PULPS.—TREATMENT AND FILLING.

BY DR. J. P. WILSON, OF BURLINGTON.

[Read before the Iowa State Dental Society.]

It is unfortunate, both for dentists and their patients, that filling teeth is, as a rule, deferred until the premonitions of pain, or tooth-ache in all its severity, admonishes the sufferer that something must be done. And it is not until the home remedies, such as smoking or chewing the "weed," applying Wizzard or St. Jacob's Oil, etc., are found to be wanting in their efficiency, producing only temporary relief, that the services of a dentist are required.

After dosing the pulp of a tooth in that manner for any length of time, I am of the opinion that arsenic is indicated, and devitalization necessary. I have come to this conclusion after years of painful experience, for it has only been a few years since I, with many of you, "caught the spirit of the times," which said never destroy, but always restore to health, cap or protect the exposed pulp, fill the tooth, and all will be well. How many of us were delighted with this fascinating theory, and mounting the hobby have ridden it to death.

Some of the eminent members of our profession have gone still farther, and said, "If half the nerve of a tooth be dead and half of it alive, amputate the disorganized part, heal the wound, then fill the tooth, and the remaining fibers will still live and sustain the organ." Such a theory is, in my humble opinion, nonsense and supreme folly. The gentlemen who have advocated this theory have spoken of it as a most delicate operation in surgery; but who cares for a fine spun theory, when experience tells us that in practice it fails.

Two distinguished surgeons were once discussing a certain capital operation in surgery. Each one had his peculiar method of operating, and clung to his theory with great tenacity. One had performed the operation a score of times, and had been successful in saving the lives of fifty per cent. of the number operated upon. The other rejoined, "I have performed the operation according to my method a hundred times." "Indeed," said his opponent, "but how many of your patients have died?" "Oh," said he, "they have all died, but the operation is very beautiful." And so it is with this beautiful operation of amputating the nerve, and of "capping" badly exposed pulps; not one per cent. of them will live for two years. They may slumber along for a time, but sooner or later there will come a mighty awakening. You may not hear from your patient; he may endure a few days suffering, rather than return to you for further treatment. His tooth is too sore, and his agony too great to allow any one to touch it. He suffers on until nature establishes an outlet for the accumulated gases and pus. Relief from pain has come, but a nuisance has been created, and an ever-flowing fountain of corruption is the result.

But, be it far from me to make a wholesale denunciation of the practice of capping nerves. I am an advocate of the theory, when the *conditions* are favorable. I am opposed to it when they are unfavorable. When one man says it is a "crime" to destroy the nerve of a tooth under any circumstances, and another says it should always be destroyed, if at all exposed, or has given any trouble whatever, I take my stand midway between them, and say, "Here is the safe ground."

The whole profession has been swaying from one extreme to the other, for the last decade, and in this movement, like every other, a few leading spirits are influencing the masses, and forming opinions for them.

I would not throw a single reflection on the able men who are engaged in the literary work of our profession, but I would suggest that the man who writes most, and practices least, may not be as correct in his conclusions as the intelligent observer who is found day after day at the chair, wielding his surgical instruments instead of his pen. I say this with reluctance, for I am proud of the literary talent in our profession, and wish we had more. But is there not danger in accepting the conclusions of those writers, without thought and investigation. Of course, it is important that we become familiar with written theory and practice, but after that, "Experience is the best teacher." And then, there is a peculiar satisfaction in a practice that experience and reason tells us is right. I would that we tie our faith to no man, but think and act for ourselves.

Seven or eight years ago I capped five pulps where I now cap one. Why? Because I then had confidence in the practice. I believed what the more enthusiastic members of our profession said, that it was very wrong to kill the nerve of a tooth, yea, more, that it was malpractice to do so, and as I did not want to do anything that was very wrong, nor did I want to be guilty of malpractice. I fell in with the new theory, and came well-nigh making it a hobby. But as time passed on the visits of dissatisfied patients of other dentists, as well as my own, became more and more frequent. I have seen as many as three or four open abscesses in one mouth, giving but little, if any, physical pain, but causing an offensive breath, and a generally unhealthy condition of the oral cavity. Capping pulps indiscriminately was the cause, and this distressingly unhealthy condition the result. My faith in the practice weakened. The fault could not be in me as an operator, for I had followed the direction of the advocates and teachers of the theory, and not only so, but the evidences of their failures, also, came too frequently to my notice, to justify me in the belief that I alone was at fault. What, therefore, is the inevitable conclusion—simply this, "The theory that all exposed pulps may be saved alive, is a false theory." But what class of exposed pulps may be saved alive, and what conditions are favorable to that end, is the question for us now to consider. Where shall we draw the line? Let us see. Here is a tooth that has been aching, the pulp is badly exposed, or protected only by disorganized tooth-bone. Here is another that has been grumbling a little, thermal changes effect it. The greater portion of the pulp is protected by healthy dentine, one point of the decay has barely reached the pulp, causing only a slight exposure, there has been no tooth ache. In the first case I should not attempt to save the life of the nerve. In the second case I should not only attempt it, but expect to succeed. Again, accidental exposures of the pulp sometimes occurs, when the operator has not been sufficiently careful in observing the form of the tooth. This blunder is inexcusable, as much so, perhaps, as it would be for a surgeon to cut into an artery for want of a proper knowledge of its location. In such an exposure the hemorrhage may be quite profuse, and yet, this is one of the safest cases for capping exposed pulps. We have here a fresh wound, and no disease whatever to contend with. A little creosote will arrest the bleeding, after which cap in the usual way, and proceed at once to fill the cavity. It is scarcely necessary to dwell at length on the manner of capping nerves. Suffice it to say, all disorganized tooth-bone should be removed, the cavity should be bathed with creosote or carbolic acid, then dried immediately, after which apply the capping paste (whatever it may be) so soft that it will on the slightest pressure with a pellet of cotton or spunk adapt itself readily to the sensitive nerve and to the floor of the cavity. If it is desirable, another layer may be added in a few minutes, when the permanent filling may be introduced.

I have purposely omitted to mention the material I would use for capping pulps. The different pastes in use all have their advocates—and all possess merit, and as I believe one loses a great deal by continually experimenting, I have hesitated to recommend what some of you may have long since abandoned. But I must say, that in my hands Smith's oxychloride of zinc has been very satisfactory. I mix the powder with equal parts of the liquid and water, which makes a paste almost non-irritating.

Before leaving this part of my subject, let me say, that the first twenty-four hours after capping a pulp usually determines whether or not the operation will be a success. Let me relate a case in practice. One week ago Mrs. B. called at my office, saying, "I have a very sensitive tooth, and want it filled." On examination the pulp was found to be considerably exposed. The tooth had ached a few times when cold water, cold air, or sweets had been brought in contact with it. It was a second upper molar, decayed from its posterior proximate surface, so that access to the root canals would be exceedingly difficult, and so I tried to persuade myself that the pulp of that tooth could be saved alive. I therefore prepared the cavity with great care, capped the nerve, and filled the cavity with "gold and platina alloy." The tooth was grumbling when my patient left the office. Before leaving town she called to tell me that her tooth was now "all right, no soreness, no pain." "But," said she, "it ached tremendously the first night, and grumbled all the next day, but since then it has given me no trouble whatever. I am so glad you didn't kill the nerve." Now, what may I expect of this case. My patient is at present satisfied. I am not, for I have no more doubt about the fate of that pulp than I would had arsenic been applied. Let it alone, and it may give no further trouble for weeks or months, but the calm is like that which precedes a coming storm.

The class of exposed pulps that require devitalization have already been indicated, and it remains now to mention in detail the treatment.

It is sometimes only necessary to take a syringe, and rinse a cavity with warm water in order to expose the pulp to view. At other times the extraneous matter covering the pulp must be removed with instruments. It is important that the arsenic be applied directly in contact with the nerve. Its action is more prompt, and it causes much less pain, and frequently gives entire relief from pain. Perhaps the most convenient way of applying the nerve paste is by dipping a very small pellet of cotton but a little larger than a pin's head into the nerve paste, then place it against the exposed pulp, and confine it there with a pellet of cotton saturated with sandarac varnish. Usually twenty-four hours is sufficient time to allow it to remain, but if foreign matter separates the arsenic from the pulp a greater length of time will be required, and much more pain will follow.

When the pulp is greatly inflamed the application of arsenic does not always accomplish the desired object. Indeed, the arsenic only aggravates the case, and it is needless to renew the application until the inflammation has been allayed by palliative treatment. But to reduce the inflammation of the pulp in all cases before attempting to kill the nerve is a needless precaution, as arsenic will accomplish its work in nineteen cases out of twenty if the pulp is in an inflamed condition. The next thing after devitalization is to thoroughly extirpate the pulp. This is more easily said than done. It is my custom to thoroughly prepare the cavity for filling before attempting to remove the pulp, and in this preparation the orifice of each canal should be fully exposed to view, after which they should be reamed out with a cone-shaped instrument, so that easy access may be attained. The openings into the canals are now surrounded by the clean, white walls of the cavity, which aids greatly in the exploration of the roots, and not only so, but the debris has all been cleared away, so that no foreign matter can be introduced into the canals to engorge them, and cause future trouble. With an assortment of nerve instruments from Palmer's and Arrington's sets, I am now prepared for the task of entering the canals.

The removal of the nerve vessels from the palatine roots of the superior molars, the posterior roots of the inferior molars, the cuspids, and the incisors, is usually so simple an operation that little or no trouble is ever experienced. The canals of the bi-cuspids, also, are, as a rule, easily explored. But this cannot be said of the buccal roots of the upper molars, or the anterior roots of the lower molars, for these sometimes baffle our best efforts, and I will frankly confess that I do not always reach the apex of these roots, nor do I consider it important, if the canals are *so small* and tortuous that they cannot be traced the entire length with a fine nerve instrument. By a fine nerve instrument I do not mean one that is so small that it will pass through the apical foramen, for with such an instrument there is continual danger of wounding the peridental membrane, the irritation of which will most likely give rise to pericementitis.

Let it not be understood from what has been said, that I would license a lack of thoroughness in preparing and filling the root canals. Every canal should be emptied and cleansed that can be thoroughly filled. But many of the buccal roots of the upper molars and the anterior roots of the lower molars can only be traced half or two-thirds their length, and it is in those cases that I do not believe it important that those exceedingly small portions of the pulp should be removed, if they are bathed for a week or ten days in carbolic acid or creosote, and then embalmed with an oxychloride cement.

We are told that every vestige of the pulp must be removed, and what I have said may seem to be dangerous doctrine, but it is not at variance with common practice. In this connection I would say that gutta-percha should never be used in contact with the slightest remains of the pulp, as it has not the power of mummifying it, as has an oxychloride cement.

In twenty-four hours after the arsenic has been applied, or as soon as the nerve has been destroyed, as before stated, then cleanse the pulp canals, and fill them with cotton moistened with creosote or carbolic acid, then fill the cavity with cotton saturated with sandarac varnish, which makes a perfectly air-tight filling for quite a length of time. In *one week* from the time this application is made. the patient may return for the purpose of having the tooth filled, or at least to have the canals filled.

After applying the rubber-dam, the medicated cotton in the roots should be removed, and the tooth is usually found to be in a condition to be filled, the necessary preparation having been made at the time the pulp was removed. Mix an oxychloride cement about the consistency of thick cream, put into it a few shreds of cotton as a vehicle to carry the cement, then take a nerve instrument smaller than the canal to be filled, and carry the shreds of cotton to the apex of the root, and leave it there embedded in the cement. Filling the balance of the canal is an easy matter. Everything depends upon the introduction of the first filling material. There should be the fewest shreds of cotton that can possibly be used for introducing the first cement; the air can then pass out, as the filling passes in. The tooth may be filled permanently at this sitting, or not, as the operator may deem best.

Before leaving this subject I should like to refer very briefly to the teachings of some of our prominent operators upon this subject. One writer suggests that "when arsenic is applied to the nerve it is a very good plan to leave it there until the death of the pulp is indicated by the tooth becoming sore." This I regard as a very dangerous practice. This condition does not only indicate that the pulp is dead, but that the effects of the arsenic have reached the peri-dental membrane. The pulp and this delicate membrane being continuous, why may it not receive a permanent injury in that way, as well as from the escape of arsenic at the cervical floor of the cavity—a thing that we are all careful to avoid when applying arsenic to a nerve. It is a much safer plan to remove the arsenic in twenty-four or forty-eight hours, and if there *should* be some pain in separating the nerve from its attachments at the apex of the root, the patient should understand that this is a thing to be endured in preparing a sensitive tooth for filling.

Another practice of some of our best operators is to leave a devitalized pulp until putrefaction or sloughing takes place, arguing that the entire pulp remains can be more thoroughly removed in that way than in any other; that a putrid, liquid mass can be *washed* away more thoroughly than it can be removed with instruments before disorganization takes place. There are serious objections to this method of treatment, and it should be avoided. It is easier to keep the pulp-canals pure and healthful than it is to invite putrefaction, and then by disinfection restore them to purity again. Indeed, those inaccessible canals may not be thoroughly deodorized, neither can the tubuli, when once infiltrated with foreign matter, be so easily disinfected. The dentine itself, often becomes thoroughly permeated with the confined impurities of a dead pulp. And this is another objection to leaving the cavity open, especially in the teeth of young people. The tubuli will in time take up the impurities, the tooth will become not only discolored, but impregnated with the filthy odor.

